# Sequential control of myeloid cell proliferation and differentiation by cytokine receptor-based chimeric antigen receptors

**DOI:** 10.1371/journal.pone.0279409

**Published:** 2022-12-27

**Authors:** Kyoko Nakajima, Zhongchuzi Shen, Masashi Miura, Hideto Nakabayashi, Masahiro Kawahara

**Affiliations:** 1 Laboratory of Cell Vaccine, Center for Vaccine and Adjuvant Research (CVAR), National Institutes of Biomedical Innovation, Health and Nutrition (NIBIOHN), Ibaraki-shi, Osaka, Japan; 2 Department of Chemistry and Biotechnology, Graduate School of Engineering, The University of Tokyo, Bunkyo-ku, Tokyo, Japan; Cincinnati Children’s Hospital Medical Center, UNITED STATES

## Abstract

As chimeric antigen receptor (CAR)-T cell therapy has been recently applied in clinics, controlling the fate of blood cells is increasingly important for curing blood disorders. In this study, we aim to construct proliferation-inducing and differentiation-inducing CARs (piCAR and diCAR) with two different antigen specificities and express them simultaneously on the cell surface. Since the two antigens are non-cross-reactive and exclusively activate piCAR or diCAR, sequential induction from cell proliferation to differentiation could be controlled by switching the antigens added in the culture medium. To demonstrate this notion, a murine myeloid progenitor cell line 32Dcl3, which proliferates in an IL-3-dependent manner and differentiates into granulocytes when cultured in the presence of G-CSF, is chosen as a model. To mimic the cell fate control of 32Dcl3 cells, IL-3R-based piCAR and G-CSFR-based diCAR are rationally designed and co-expressed in 32Dcl3 cells to evaluate the proliferation- and differentiation-inducing functions. Consequently, the sequential induction from proliferation to differentiation with switching the cytokine from IL-3 to G-CSF is successfully replaced by switching the antigen from one to another in the CARs-co-expressing cells. Thus, piCAR and diCAR may become a platform technology for sequentially controlling proliferation and differentiation of various cell types that need to be produced in cell and gene therapies.

## Introduction

Cell and gene therapies have become one of the major fields in medicine for curing intractable diseases [1]. Bone marrow transplantation has been successfully established by deep knowledge on hematopoietic stem cells and immune rejection due to human leukocyte antigen mismatch [2]. Furthermore, in recent years, chimeric antigen receptor (CAR)-T cell therapy has been practically applied in clinics, which is an epoch-making success in cell and gene therapies [3]. Therefore, controlling the fate of blood cells is increasingly important for clinical applications in this field.

In therapies using blood cells, in vitro amplification and subsequent differentiation of hematopoietic stem cells are indispensable. Generally, cell proliferation and differentiation are regulated by cytokine receptors, which oligomerize upon receiving the corresponding cytokines and activate downstream signal transduction cascades. For instance, c-Kit, c-Mpl, and Flt-3 are known to transduce proliferation signals in hematopoietic stem cells in response to stem cell factor, thrombopoietin, and Flt-3 ligand, respectively [4]. In addition, lineage-specific growth factors such as granulocyte-macrophage colony-stimulating factor (GM-CSF), erythropoietin, thrombopoietin, interleukin-2, -3, and -7 play a pivotal role in producing a sufficient number of progenitor and terminally differentiated blood cells [5].

However, a large amount of cytokines are required to produce a sufficient number of differentiated cells in vitro, which makes the overall process too costly for practical applications in cell and gene therapies [6]. To overcome the limitation, investigators have screened surrogate ligands that activate cytokine receptors. Antibodies [7], peptides [8], nucleic acids [9], and even small molecules [10] have been reported to function as agonists at cytokine receptors. However, since the conformation is important for activation of cytokine receptors, these agonists do not always retain the same levels of receptor activation capability as natural cytokines [11]. In addition, simple screening of receptor binders may often select antagonists that inhibit activation of cytokine receptors [12]. An alternative approach is to screen small molecules targeting signal transduction pathways. A marked example is StemRegenin 1, which is an antagonist at differentiation-inducing aryl hydrocarbon receptor and promotes expansion and maintenance of hematopoietic stem cells [13]. Although such small-molecule inhibitors are practically useful also in cell types other than hematopoietic systems (*e*.*g*. 2i culture of induced pluripotent stem cells) [14, 15], a whole signaling pathway is often blocked, which makes it difficult to regulate activation levels of signaling in a more sophisticated manner.

We have developed cytokine receptor-based chimeric antigen receptors (CARs) that mimic the activation mechanism of natural cytokine receptors [16, 17]. The ligand-binding domain of cytokine receptors was replaced with single-chain Fv (scFv), which could alter ligand specificity from cytokines to specific antigens. In our previous studies, we have successfully attained antigen-induced proliferation [18, 19], differentiation [20], migration [21, 22], and death [23, 24] using appropriate signaling domains derived from cytokine receptors. However, sequential regulation from proliferation to differentiation, which would be the most fundamental and important in cell and gene therapies for curing blood disorders, has yet to be demonstrated.

In this study, we aim to construct proliferation-inducing and differentiation-inducing CARs (piCAR and diCAR) with two different antigen specificities and express them simultaneously on the cell surface. The two antigens are non-cross-reactive and exclusively activate piCAR or diCAR. Thus, sequential induction from cell proliferation to differentiation could be controlled by switching the antigens added in the culture medium. To demonstrate this notion, a murine myeloid progenitor cell line 32Dcl3 is chosen as a model for hematopoietic cells. 32Dcl3 cells proliferate in an IL-3-dependent manner and differentiate into granulocytes when cultured in the presence of G-CSF [25]. Therefore, piCAR and diCAR are constructed using the IL-3 receptor and G-CSF receptor with scFv having different antigen specificities. We investigate whether the piCAR and diCAR could sequentially induce proliferation and differentiation according to the addition of the corresponding specific antigens when co-expressed in 32Dcl3 cells.

## Materials and methods

### Plasmid construction

A lentiviral plasmid pL-SIN-EF1α-EGFP (Addgene #21320) was modified to encode one of the CAR genes and an internal ribosomal entry site (IRES)-antibiotic resistance gene cassette instead of the originally encoded EGFP gene. The resultant plasmids were pL-SIN-EF1α-V5-S(M)-IL3Rα-IRES-Neo^R^, pL-SIN-EF1α-Flag-S(M)-IL3Rβc-IRES-Blast^R^, and pL-SIN-EF1α-HA-S(U)-GCSFR-IRES-Puro^R^, in which an immunoglobulin κ chain-derived signal sequence and the tag sequence were appended at the N-terminus of each CAR gene for enabling cell surface expression and the detection with immunostaining and flow cytometric analysis. The piCAR encodes anti-fluorescein scFv clone 4M5.3 (S(M)) [26], the extracellular D2 domain of EpoR, the transmembrane and intracellular domain of IL3Rα or IL3Rβc, whereas the diCAR encodes anti-dinitrophenol scFv clone U7.6 (S(U)) [27], the extracellular D2 domain of EpoR, the transmembrane and intracellular domain of GCSFR. The amino acid sequences of piCAR and diCAR are summarized in (S1-S3 Figs in S1 File).

### Cell culture

Murine IL-3-dependent myeloid progenitor 32Dcl3 cells (RIKEN Cell Bank #RCB1377, Ibaraki, Japan) were subcloned and cultured in RPMI1640 medium (Nacalai Tesque, Kyoto, Japan) supplemented with 10% fetal bovine serum (Sigma-Aldrich, St. Louis, MO) and 1 ng/ml murine IL-3 (Thermo Fisher Scientific, Waltham, MA). For lentiviral packaging, 293T cells were cultured in Dulbecco’s modified Eagle’s medium (DMEM) (Nacalai Tesque) supplemented with 10% fetal bovine serum.

### Lentiviral transduction

293T cells were transfected with the mixture of each lentiviral plasmid and packaging plasmids (pCMV-dR8.91 and pCMV-VSV-G) using Lipofectamine LTX (Thermo Fisher Scientific). Two days later, the lentiviral supernatant was filtrated with a 0.45 μm PVDF filter and mixed with 32Dcl3 cells (1 x 10^5^ cells) in a 24-well plate in the presence of 1 ng/ml IL-3 and 10 μg/ml protamine sulfate (Fujifilm Wako Pure Chemical, Osaka, Japan). Two days later, the transduced cells were harvested and selected with antibiotics. The antibiotics concentrations used were 400 μg/ml G418 (Calbiochem, La Jolla, CA), 20 μg/ml blasticidin (Kaken Pharmaceutical, Tokyo, Japan), and 2 μg/ml puromycin (Sigma-Aldrich).

### Cell surface immunostaining

The overall protocol for cell surface immunostaining was described previously [28]. In brief, cells were collected and resuspended with PBS containing a mouse primary antibody (1:200 dilution). The cells were incubated on ice for 30 min, washed twice with PBS, and resuspended with PBS containing a phycoerythrin (PE)-conjugated secondary antibody (1:200 dilution). The cells were incubated on ice for 30 min, washed twice with PBS, and resuspended with PBS. The cells were analyzed by a FACSCalibur flow cytometer (BD Biosciences, Lexington, KY). FlowJo software (BD Biosciences) was used to generate histograms and calculate median fluorescence intensity. The antibodies used are summarized in (S4 Fig in S1 File).

### Cell proliferation assay

The experimental procedures for cell proliferation assays were described previously [28]. Cells were washed with PBS twice and inoculated into 24-well plates at 5 x 10^4^ cells/ml in the culture medium with various concentrations of FL-BSA (Sigma-Aldrich) or 1 ng/ml IL-3. On day 2–4, viable cell number was counted by flow cytometry or estimated using Cell Counting Kit-8 (Dojindo Laboratories, Kumamoto, Japan) by measuring 450 nm absorbance with GloMax Discover Microplate Reader (Promega, Madison, WI).

### Cell differentiation assay

Cells were washed with PBS twice and inoculated into 24-well plates at 1 x 10^5^ cells/ml in the culture medium with 1 ng/ml IL-3, 10 ng/ml G-CSF (R&D Systems, Minneapolis, MN), 1 μg/ml FL-BSA, or 0.1 μg/ml DNP-BSA (Thermo Fisher Scientific). On day 5 or 6, the cells were stained with 50 μl PBS containing PE-conjugated rat anti-mouse CD11b (1:100 dilution; BD Biosciences) or PE rat IgG2b κ isotype control (1:100 dilution; BD Biosciences). The cells were incubated on ice for 30 min, washed twice with PBS, and resuspended with 200 μl PBS. The cells were analyzed by the FACSCalibur flow cytometer.

For May-Grünwald-Giemsa staining, the cells cultured in each ligand for 5 days were washed by PBS once and fixed on glass slides with methanol for 90 sec. The cells on the glass slides were immersed in May-Grünwald staining solution (Sigma-Aldrich) for 5 min, immersed in PBS for 3 min, immersed in Giemsa staining solution (Sigma-Aldrich) for 15 min, and rinsed in Milli-Q water twice. The stained cells were photographed with EVOS XL Core Cell Imaging System (Thermo Fisher Scientific).

### Signal transduction analysis

The experimental procedures for cell stimulation and subsequent western blotting were described previously [28]. Briefly, cells maintained with IL-3 or FL-BSA were cultured in the medium with neither IL-3 nor FL-BSA for 6 h. The cells were stimulated with 1 ng/ml IL-3, 10 ng/ml G-CSF, 1 μg/ml FL-BSA, or 0.1 μg/ml DNP-BSA at 37°C for 15 min. The cell lysates were prepared and analyzed by Western blotting. The specific proteins transferred on nitrocellulose membranes were probed with rabbit primary antibodies and HRP-conjugated secondary antibody. The antibodies used are summarized in (S5 Fig in S1 File).

### Statistical analysis

The original data were acquired by performing experiments with three biological replicates. The means of two groups were compared by Student’s *t*-test, whereas the means of multiple groups were compared by one-way ANOVA followed by post-hoc Bonferroni’s multiple-comparison test. Statistical significance is indicated as asterisks.

## Results

### Design of piCAR and diCAR

**Fig 1:**
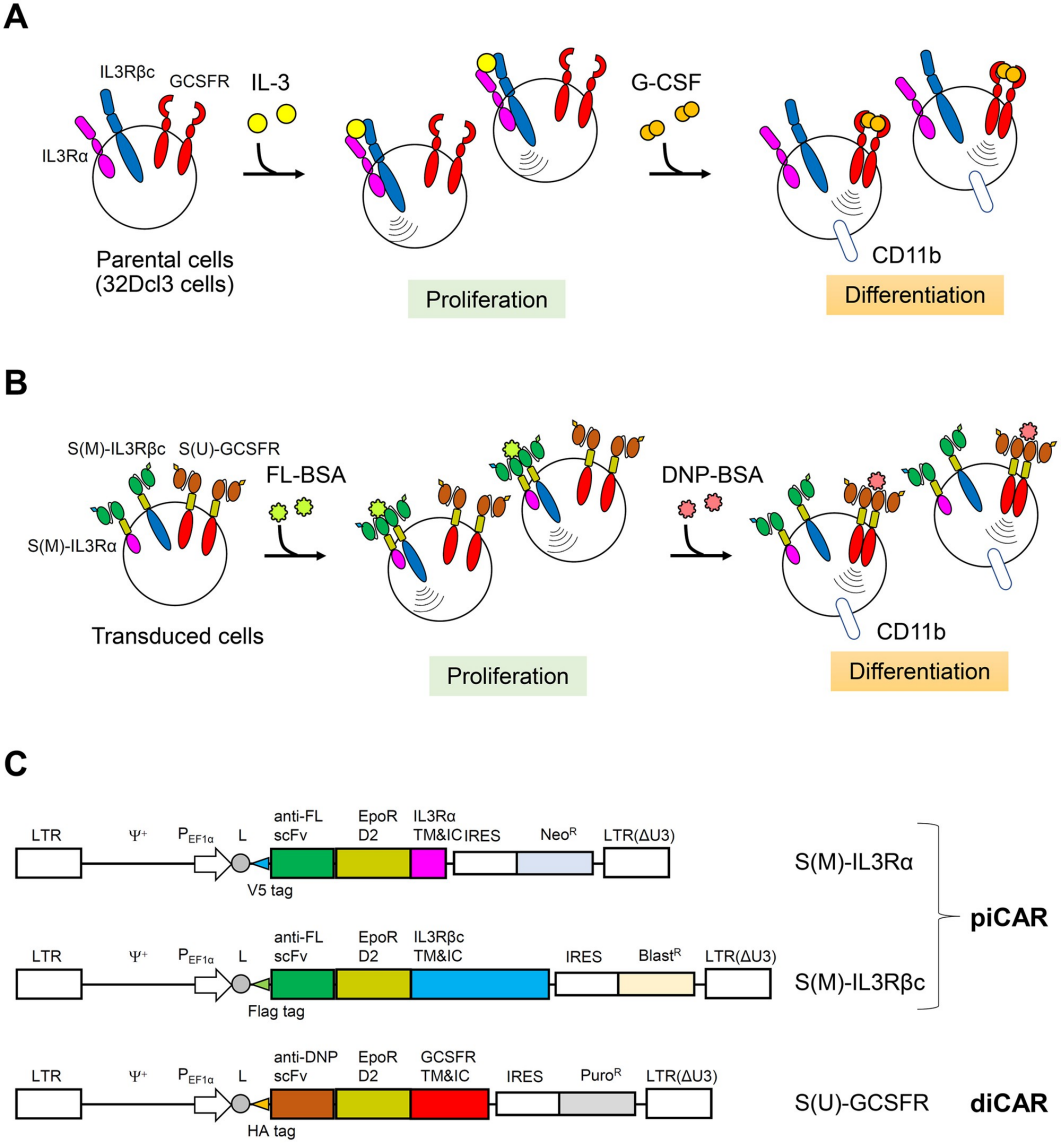
Design of piCAR and diCAR. (A) Schematic illustration for proliferation and differentiation of parental 32Dcl3 cells. (B) Antigen-dependent sequential control of proliferation and differentiation of the cells transduced with FL-specific piCAR (S(M)-IL3Rα and S(M)-IL3Rβc) and DNP-specific diCAR (S(U)-GCSFR). (C) Construction of the lentiviral expression vectors. LTR, long-terminal repeat; Ψ^+^, lentiviral packaging signal; P_EF1α_, EF1α promoter; L, leader sequence derived from immunoglobulin κ chain; TM&IC, transmembrane and intracellular domains; IRES, internal ribosomal entry site.

In this study, we aimed to develop proliferation-inducing and differentiation-inducing chimeric antigen receptors (piCAR and diCAR, respectively) with different antigen specificities for sequential control of cell proliferation to differentiation. A murine myeloid cell line 32Dcl3 proliferates in an interleukin-3 (IL-3)-dependent manner (Fig 1A). Therefore, the transmembrane and intracellular domains of IL-3 receptor (IL-3R) were chosen as the signaling domain of piCAR. IL-3R consists of an IL-3-specific α subunit and a common β subunit (βc) that is shared by IL-3R, granulocyte-macrophage colony-stimulating factor receptor (GM-CSFR), and interleukin-5 receptor (IL-5R) [29]. When IL-3 binds to the receptor, α and βc subunits form a heterodimer, which activates the intracellular signaling pathways such as Ras/MAPK and PI3K/Akt pathways, and induces cell proliferation. The extracellular domain of piCAR contains a single-chain Fv antibody clone 4M5.3, which specifically binds to fluorescein (FL), and the D2 domain of erythropoietin receptor (EpoR). The 4M5.3 scFv was affinity-matured by yeast display from an antibody clone 4-4-20 derived from a murine hybridoma [26]. The EpoR D2 domain was included in the CARs because our previous studies had already demonstrated that CARs with the EpoR D2 domain and diverse cytoplasmic signaling domains are functional [18–22, 24, 28]. The transmembrane and intracellular domains derived from IL-3Rα and βc chain were utilized to create a pair of piCAR chains, named S(M)-IL3Rα and S(M)-IL3Rβc, respectively (Fig 1B). To enable detection of cell surface expression of the piCAR chains by flow cytometry, V5 and Flag tags were appended to the N-terminus of S(M)-IL3Rα and S(M)-IL3Rβc, respectively.

32Dcl3 cells differentiate into granulocytes by G-CSF (Fig 1A). Therefore, the transmembrane and intracellular domains of the G-CSF receptor were used as the signaling domain of diCAR. An anti-2,4-dinitrophenol (DNP) single-chain Fv antibody clone U7.6, which was originally derived from a murine hybridoma [27], and the EpoR D2 domain were utilized as the extracellular domain of diCAR, named S(U)-GCSFR (Fig 1B). An HA tag was appended to the N-terminus of S(U)-GCSFR to facilitate detection on the cell surface. In our previous study, we utilized the same anti-FL and anti-DNP scFv clones to demonstrate that these scFvs are non-cross-reactive to the other antigen [28]. To establish 32Dcl3 variants co-expressing S(M)-IL3Rα, S(M)-IL3Rβc, and S(U)-GCSFR, lentiviral vectors encoding each of these CAR genes were constructed (Fig 1C). To secure expression of all the three CAR genes in transduced cells, different antibiotic resistance markers were employed for the three vectors encoding the CAR genes.

### piCAR induces cell proliferation in response to FL-BSA

**Fig 2:**
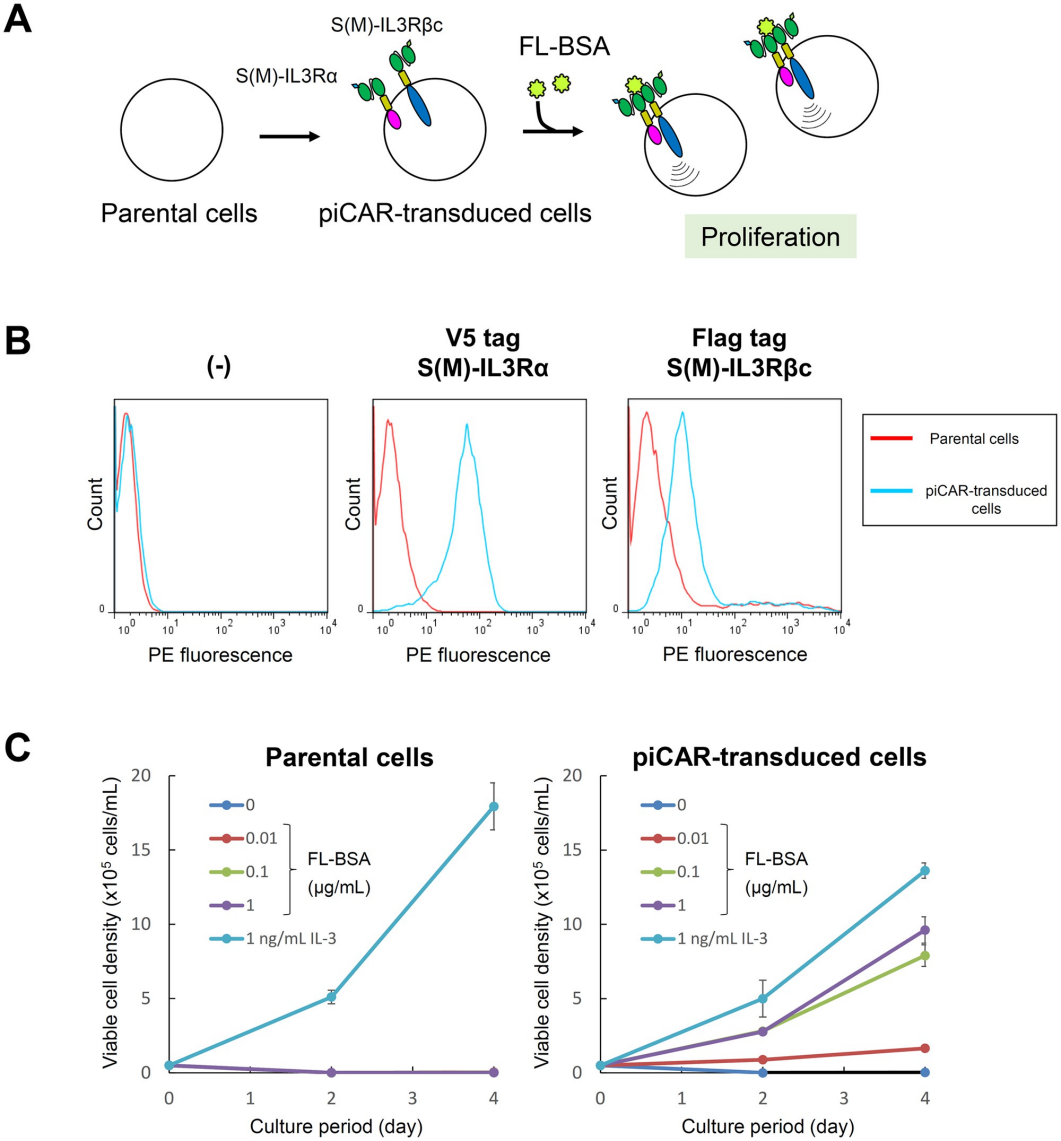
Characterization of piCAR-transduced cells. (A) Schematic illustration for establishment of piCAR-transduced cells. (B) Cell surface immunostaining of S(M)-IL3Rα and S(M)-IL3Rβc. Parental 32Dcl3 cells (red line) and piCAR-transduced cells (light blue line) were stained with each of anti-tag primary antibodies or without any primary antibodies (-), and subsequently stained with a phycoerythrin (PE)-labeled secondary antibody. Histograms are represented as PE fluorescence (x axis) versus cell count (y axis). (C) Cell proliferation assay. Cells were cultured for the indicated days in the medium with no factor, various concentrations of FL-BSA, or 1 ng/ml IL-3. Viable cell densities were measured by flow cytometry. The data are represented as mean ± SD (n = 3, biological replicates).

We firstly tested whether piCAR could induce cell proliferation in response to a specific antigen. 32Dcl3 cells were transduced with the lentiviral vectors encoding S(M)-IL3Rα and S(M)-IL3Rβc, followed by antibiotics selection of stably piCAR-transduced cells (Fig 2A). The cell surface expression of S(M)-IL3Rα and S(M)-IL3Rβc was confirmed by immunostaining the cells with each of anti-tag primary antibodies and a phycoerythrin (PE)-labeled secondary antibody and measuring the PE fluorescence by flow cytometry (Fig 2B). As a result, the peak shift of the PE fluorescence was clearly observed for both CARs, indicating that S(M)-IL3Rα and S(M)-IL3Rβc were expressed on the surface of the transduced cells.

To verify whether S(M)-IL3Rα and S(M)-IL3Rβc could induce cell proliferation in response to the target antigen FL-conjugated bovine serum albumin (FL-BSA), the cells were washed to remove IL-3 from the culture medium and switched to a FL-BSA-containing culture medium, leading to cell proliferation. Therefore, the cells were subjected to a cell proliferation assay, in which the cells were cultured with IL-3 or various FL-BSA concentrations (Fig 2C). As a result, the transduced cells expressing the two CAR chains showed FL-BSA concentration-dependent proliferation, although the cell proliferation levels with FL-BSA were lower than that with IL-3. On the contrary, the parental 32Dcl3 cells showed no proliferative response to all FL-BSA concentrations. These results demonstrate that S(M)-IL3Rα and S(M)-IL3Rβc function as piCAR.

### diCAR induces granulocytic differentiation in response to DNP-BSA

**Fig 3:**
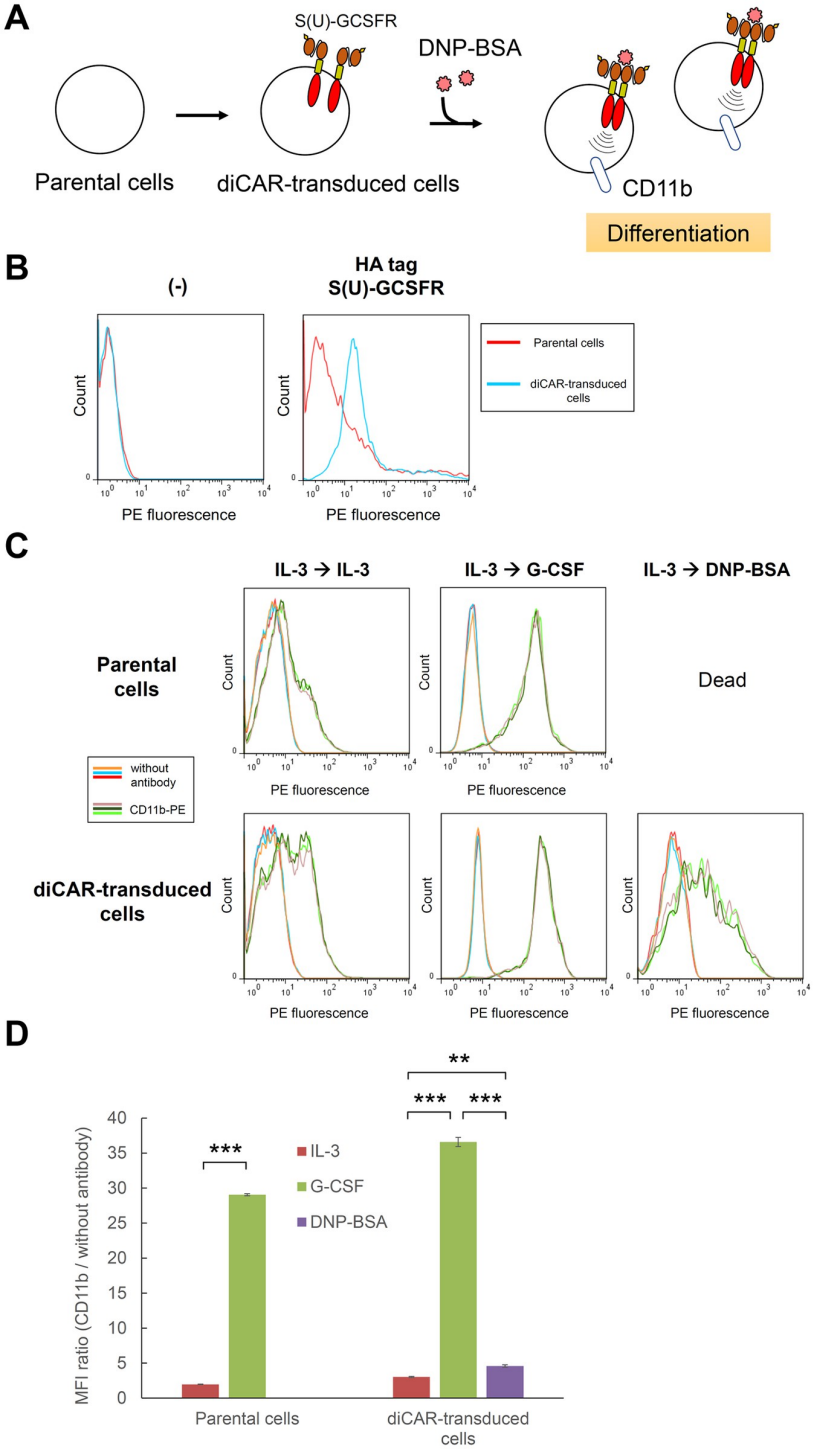
Characterization of diCAR-transduced cells. (A) Schematic illustration for establishment of diCAR-transduced cells. (B) Cell surface immunostaining of S(U)-GCSFR. Parental 32Dcl3 cells (red line) and diCAR-transduced cells (light blue line) were stained with an anti-tag primary antibody or without the primary antibody (-), and subsequently stained with a PE-labeled secondary antibody. Histograms are represented as PE fluorescence (x axis) versus cell count (y axis). (C) Cell differentiation assay. Cells precultured with 1 ng/ml IL-3 were washed and cultured with 1 ng/ml IL-3, 10 ng/ml G-CSF, or 0.1 μg/ml DNP-BSA. On day 6, the cells were stained without antibody or with PE-labeled anti-mouse CD11b. Parental 32Dcl3 cells were used as controls. Histograms in each panel are represented for three biological replicates stained without antibody (orange, light blue, and red lines) and for those stained with CD11b-PE (flaxen, dark green, and light green lines). (D) Median fluorescence intensities (MFI) were calculated for the histograms presented in (C), and MFI ratios (CD11b / without antibody) were shown as mean ± SD (n = 3, biological replicates). Student’s *t*-test (for parental cells) and one-way ANOVA followed by post-hoc Bonferroni’s multiple-comparison test (for diCAR-transduced cells) were used for comparing the means of the groups. ***p < 0.001; **p < 0.01.

We next examined whether diCAR could induce granulocytic differentiation in response to a specific antigen. 32Dcl3 cells were transduced with the lentiviral vector encoding S(U)-GCSFR, followed by antibiotic selection to establish diCAR-transduced cells (Fig 3A). To confirm the cell surface expression of S(U)-GCSFR, immunostaining was performed using anti-tag primary antibody and PE-labeled secondary antibody (Fig 3B). Flow cytometric analysis showed an apparent peak shift of the PE fluorescence in the diCAR-transduced cells, demonstrating the cell surface expression of S(U)-GCSFR.

Then, we investigated whether the cells could differentiate into granulocytes with DNP-conjugated BSA (DNP-BSA), which is a specific oligomeric antigen toward S(U)-GCSFR. The cells precultured with IL-3 were divided into three conditions, in which the cells were cultured with the original proliferation factor (IL-3), the differentiation factor (G-CSF), or the S(U)-GCSFR-mediated putative differentiation factor (DNP-BSA). The expression level of CD11b, which is a granulocytic differentiation marker, was measured for the cells by immunostaining and subsequent flow cytometric analysis (Fig 3C and 3D). Consequently, the transduced cells expressed higher levels of CD11b when cultured with G-CSF or DNP-BSA than with IL-3. CD11b was expressed even in IL-3-cultured cells, suggesting an incomplete nature of endogenous IL-3R for maintaining an undifferentiated state of 32Dcl3 cells. The expression levels of CD11b in DNP-BSA-cultured cells were lower than those in G-CSF-cultured cells, indicating that S(U)-GCSFR-mediated differentiation was less efficient than endogenous G-CSFR-mediated one. In contrast, parental 32Dcl3 cells were not able to even survive in the DNP-BSA condition, which clearly indicated that the DNP-BSA-mediated differentiation in the transduced cells was derived from diCAR.

To further demonstrate the functionality of diCAR, the cells cultured in each ligand were subjected to May-Grünwald-Giemsa staining to visualize the morphologies via microscopy (S6 Fig in S1 File). Intriguingly, G-CSF and DNP-BSA induced cells with segmented nuclei in diCAR-transduced cells, which is a hallmark of granulocytic differentiation. In contrast, the proliferation factor IL-3 did not induce cells with segmented nuclei in both parental and diCAR-transduced cells. Taken together, the results demonstrate that S(U)-GCSFR functions as granulocyte-inducing diCAR.

### Co-expression of piCAR and diCAR induces proliferation and differentiation sequentially

**Fig 4:**
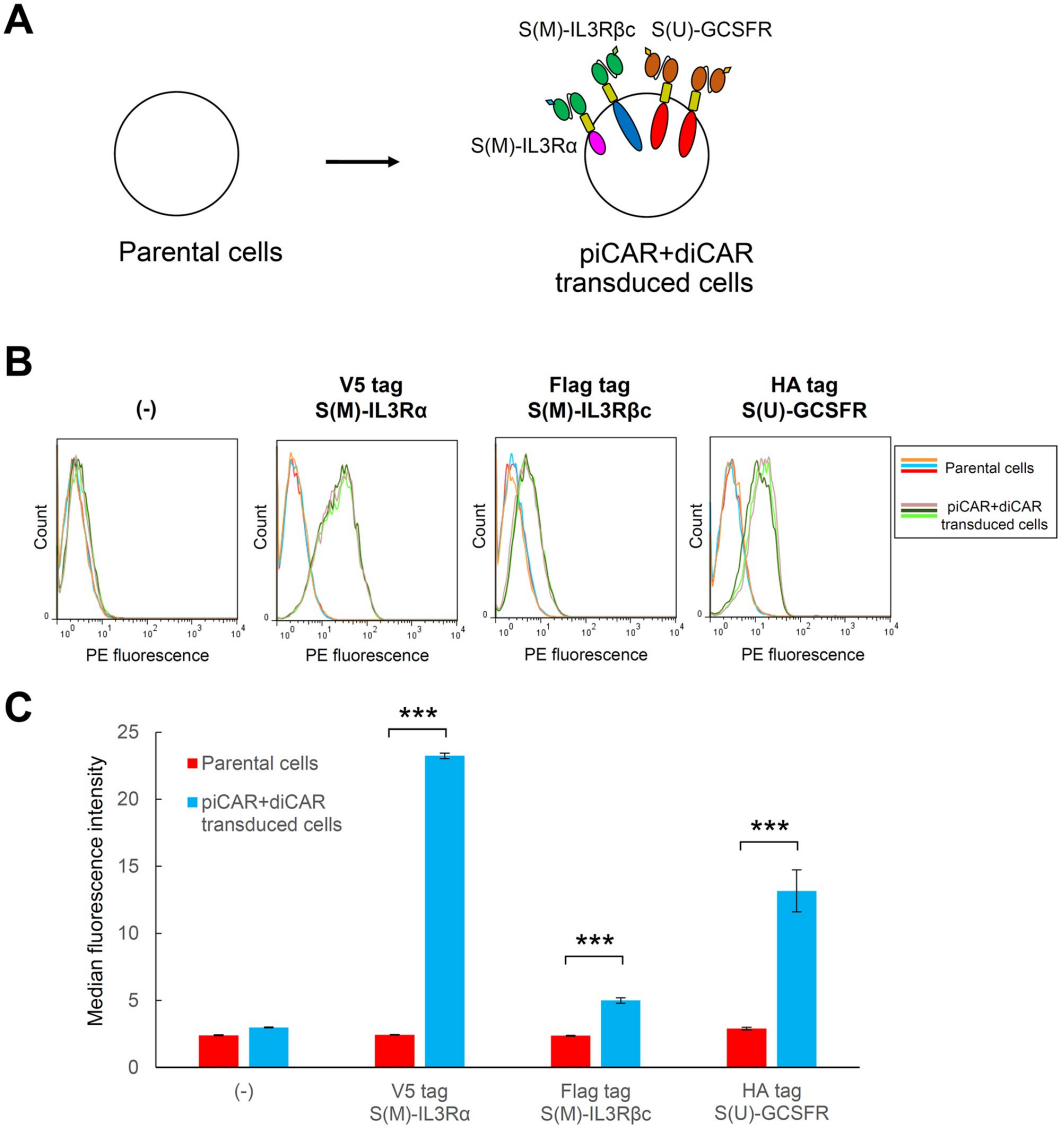
Establishment of transduced cells co-expressing piCAR and diCAR. (A) Schematic illustration for establishment of transduced cells co-expressing piCAR and diCAR. (B) Cell surface immunostaining of the CARs. Three replicates of parental 32Dcl3 cells (orange, light blue, and red lines) and those of the cells co-transduced with S(M)-IL3Rα, S(M)-IL3Rβc, and S(U)-GCSFR (flaxen, dark green, and light green lines) were stained with each of anti-tag primary antibodies or without any primary antibodies (-), and subsequently stained with a PE-labeled secondary antibody. Histograms are represented as PE fluorescence (x axis) versus cell count (y axis). (C) Median fluorescence intensities were calculated for the histograms presented in (B) and shown as mean ± SD (n = 3, staining replicates). Student’s *t*-test was used for comparing the means of the groups. ***p < 0.001.

Since piCAR and diCAR were demonstrated to be functional in 32Dcl3 cells, we investigated whether co-expression of piCAR and diCAR could sequentially induce proliferation and granulocytic differentiation. 32Dcl3 cells were transduced with all of the three lentiviral vectors encoding S(M)-IL3Rα, S(M)-IL3Rβc, and S(U)-GCSFR, followed by selection of stably transduced cells with a cocktail of three antibiotics (Fig 4A). Immunostaining with anti-tag antibodies and subsequent flow cytometric analysis revealed that although the expression of S(M)-IL3Rβc was relatively weak, the peak shift of the PE fluorescence was observed for all tags, indicating that all three CAR chains were expressed on the surface of the transduced cells (Fig 4B and 4C).

**Fig 5:**
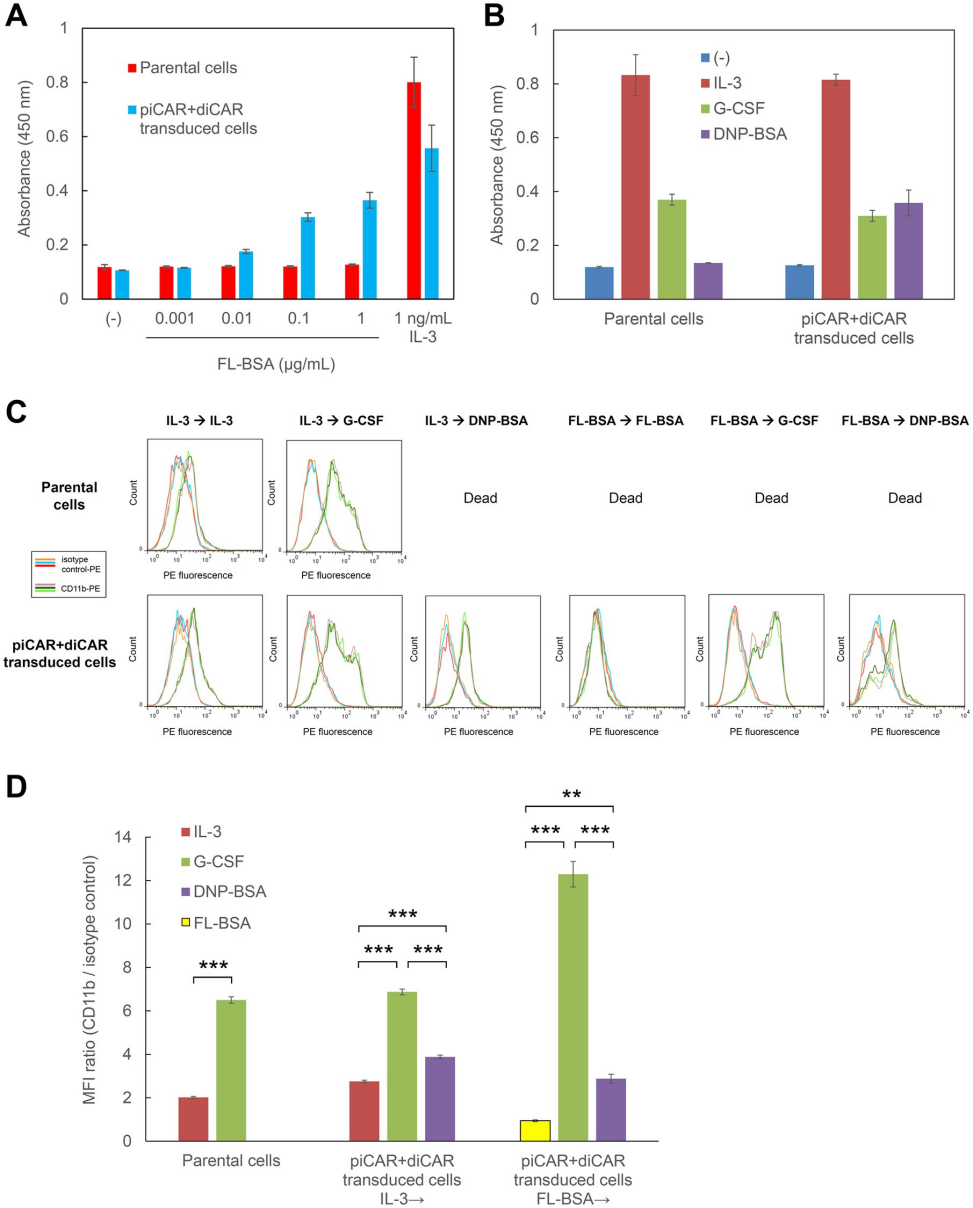
piCAR and diCAR induce proliferation and granulocytic differentiation. (A) Cell proliferation assay to examine piCAR-mediated proliferation. Cells were cultured for 3 days in the medium with no factor (-), various concentrations of FL-BSA, or 1 ng/ml IL-3. Cell proliferation levels were estimated with a colorimetric assay by measuring absorbance at 450 nm. The data are represented as mean ± SD (n = 3, biological replicates). (B) Cell proliferation arrest induced by diCAR. Cells were inoculated at 2 x 10^5^ cells/ml and cultured for 3 days in the medium with no factor (-), 1 ng/ml IL-3, 10 ng/ml G-CSF, or 0.1 μg/ml DNP-BSA. Cell proliferation levels were estimated with a colorimetric assay by measuring absorbance at 450 nm. The data are represented as mean ± SD (n = 3, biological replicates). (C) Cell differentiation assay. Cells precultured with 1 ng/ml IL-3 or 1 μg/ml FL-BSA were washed and cultured with 1 ng/ml IL-3, 1 μg/ml FL-BSA, 10 ng/ml G-CSF, or 0.1 μg/ml DNP-BSA. On day 5, the cells were stained with PE-labeled rat IgG2b κ isotype control or PE-labeled anti-mouse CD11b. Parental 32Dcl3 cells were used as controls. Histograms in each panel are represented for three biological replicates stained with isotype control-PE (orange, light blue, and red lines) and for those stained with CD11b-PE (flaxen, dark green, and light green lines). (D) Median fluorescence intensities (MFI) were calculated for the histograms presented in (C), and MFI ratios (CD11b / isotype control) were shown as mean ± SD (n = 3, biological replicates). Student’s *t*-test (for parental cells) and one-way ANOVA followed by post-hoc Bonferroni’s multiple-comparison test (for piCAR+diCAR transduced cells) were used for comparing the means of the groups. ***p < 0.001; **p < 0.01.

To examine whether piCAR could induce cell proliferation in response to the target antigen FL-BSA, a cell proliferation assay was performed, in which the cells were cultured with IL-3 or various FL-BSA concentrations (Fig 5A). In line with the results obtained in only piCAR-expressing cells in Fig 2C, the transduced cells co-expressing piCAR and diCAR proliferated in FL-BSA concentration-dependent manner, whereas the parental 32Dcl3 cells did not respond to FL-BSA. In addition, we also performed another cell proliferation assay to investigate whether diCAR could drive differentiation through proliferation arrest in response to DNP-BSA, which was compared to G-CSF-mediated proliferation arrest, IL-3-mediated cell proliferation, and no ligand-mediated cell death (Fig 5B). As expected, DNP-BSA and G-CSF induced similar levels of proliferation arrest in the transduced cells co-expressing piCAR and diCAR, whereas the parental 32Dcl3 cells responded only to G-CSF and did not respond to DNP-BSA.

To investigate whether the transduced cells co-expressing piCAR and diCAR could differentiate into granulocytes with the specific antigen DNP-BSA, the cells precultured with IL-3 were divided into three conditions, in which the cells were cultured with the original proliferation factor (IL-3), the differentiation factor (G-CSF), or the diCAR-mediated putative differentiation factor (DNP-BSA). In addition, the cells precultured with FL-BSA were divided into three conditions, in which the cells were cultured with the original proliferation factor (FL-BSA), the differentiation factor (G-CSF), or the diCAR-mediated putative differentiation factor (DNP-BSA). The expression level of CD11b was measured by immunostaining and flow cytometry (Fig 5C and 5D). As a result, G-CSF and DNP-BSA induced higher levels of CD11b than IL-3 and FL-BSA, regardless of the preculture conditions. Consistent with the results obtained in only diCAR-expressing cells in Fig 3C, DNP-BSA induced lower expression levels of CD11b than G-CSF, which further confirmed that diCAR transduced less efficient differentiation signals than endogenous G-CSFR. Again, the continuous culture with IL-3 induced expression of CD11b, suggesting an incomplete capability of IL-3 to inhibit differentiation of 32Dcl3 cells. Of note, the continuous culture with FL-BSA did not induce expression of CD11b, indicating that piCAR may be superior to endogenous IL-3R in terms of keeping the cells in an undifferentiated state. As expected, parental 32Dcl3 cells were dead in the FL-BSA and DNP-BSA conditions. Taken together, these results demonstrate that piCAR and diCAR can sequentially control proliferation and granulocytic differentiation of 32Dcl3 cells in response to their specific antigens.

### Signaling analysis revealed a distinct signature of signaling between piCAR and diCAR

**Fig 6:**
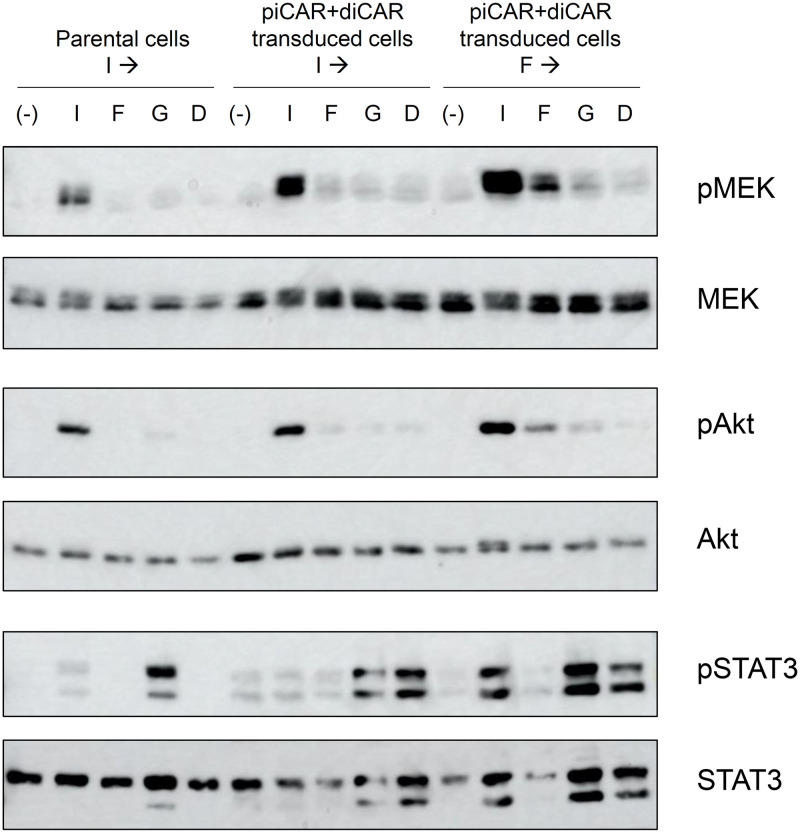
Signaling analysis revealed a distinct signature of signaling between piCAR and diCAR. Parental 32Dcl3 cells precultured with 1 ng/ml IL-3 (Parental cells I→) and the transduced cells co-expressing piCAR and diCAR precultured with 1 ng/ml IL-3 (piCAR+diCAR transduced cells I→) or 1 μg/ml FL-BSA (piCAR+diCAR transduced cells F→) were unstimulated (-) or stimulated with 1 ng/ml IL-3 (I), 1 μg/ml FL-BSA (F), 10 ng/ml G-CSF (G), or 0.1 μg/ml DNP-BSA (D). Phospho-MEK, whole MEK, phospho-Akt, whole Akt, phospho-STAT3, and whole STAT3 were detected using corresponding primary antibodies. Uncropped blot images are provided as S7 Fig in S1 File.

To compare the signaling properties induced by the CARs with those induced by endogenous IL-3R and G-CSFR, the phosphorylation levels of the signaling molecules were verified by Western blotting. We chose MEK, Akt, and STAT3 for detection of signaling via the Ras/MAPK, PI3K/Akt, and JAK/STAT pathways, respectively. Parental 32Dcl3 cells, the piCAR- and diCAR-co-transduced cells precultured with IL-3, and those precultured with FL-BSA were washed, cultured devoid of these original proliferation factors, and stimulated with IL-3, FL-BSA, G-CSF, or DNP-BSA to examine the phosphorylation levels of MEK, Akt, and STAT3 in Western blotting (Fig 6).

As a result, MEK and Akt were strongly phosphorylated by IL-3 and weakly phosphorylated by G-CSF in both parental and transduced cells. Intriguingly, MEK and Akt were weakly phosphorylated by FL-BSA and hardly phosphorylated by DNP-BSA in the transduced cells, whereas these signaling molecules were not phosphorylated by FL-BSA and DNP-BSA in the parental cells.

Interestingly, STAT3 showed an opposing phosphorylation property compared to MEK and Akt. STAT3 was strongly phosphorylated by G-CSF and weakly phosphorylated by IL-3 in both parental and transduced cells. This is consistent with previous literature that endogenous G-CSFR recruits STAT3 and activate downstream signaling to induce granulocytic differentiation [30]. As expected, STAT3 was substantially phosphorylated by DNP-BSA and hardly phosphorylated by FL-BSA in the transduced cells, whereas STAT3 was not phosphorylated by FL-BSA and DNP-BSA in the parental cells. These results demonstrate that the CARs induced similar signaling characteristics but weaker intensities of signaling compared to the corresponding endogenous receptors.

## Discussion

In this study, IL-3R-based piCAR and G-CSFR-based diCAR were rationally designed and co-expressed in myeloid cells to evaluate the proliferation- and differentiation-inducing functions. S(M)-IL3Rα and S(M)-IL3Rβc were constructed as piCAR with a FL-specific scFv and IL-3Rα/βc as the antigen recognition and signaling domains, respectively. In addition, S(U)-GCSFR was constructed as diCAR with a DNP-specific scFv and G-CSFR as the antigen recognition and signaling domains, respectively. When the cells expressing these CARs were cultured with FL-BSA, cell proliferation was successfully induced. In contrast, when the cells expressing these CARs were cultured with DNP-BSA, the expression of the granulocytic differentiation marker CD11b significantly increased, showing that granulocytic differentiation was successfully induced. Furthermore, the sequential induction from proliferation to differentiation with switching the cytokine from IL-3 to G-CSF was successfully replaced by switching the antigen from FL-BSA to DNP-BSA in the transduced cells.

The signaling analysis revealed that the activation levels of MEK, Akt, and STAT3 were weaker with the antigen stimulation (FL-BSA and DNP-BSA) than with the corresponding cytokine stimulation (IL-3 and G-CSF, respectively). This might reflect the difference in signaling efficiency due to the difference in the expression levels of the receptors and/or the difference in the conformations between the endogenous receptors and the CARs. One may further optimize the CAR constructs to get closer to the endogenous receptors.

In this study, we succeeded in sequentially controlling proliferation and granulocytic differentiation of myeloid progenitor cells, which are important for the treatment of hematopoietic diseases. As clearly shown by the clinical success of tumor-eradicating CAR-T cells, cell and gene therapies would be promising as a next-generation medical treatment that could outperform existing treatments. It would open a new scheme of cell and gene therapies if CARs could control various cell fates in various host cells to cure intractable diseases in vivo. Therefore, challenges for the future may be to generalize the concept of piCAR and diCAR to attain proliferation and differentiation of various cell types, with which the concept will be recognized as a platform for cell and gene therapies.

## Supporting information

S1 File (PDF)
