# Supplementary material for: Sequential control of myeloid cell proliferation and differentiation by cytokine receptor-based chimeric antigen receptors
Source: PLoS One. 2022 Dec 27;17(12):e0279409. doi: 10.1371/journal.pone.0279409 (PMC9794043; doi:10.1371/journal.pone.0279409)
Supplement: S1 File — (PDF) [file pone.0279409.s001.pdf]

Supporting Information

Sequential control of myeloid cell proliferation and differentiation by cytokine receptor-based chimeric antigen receptors

Kyoko Nakajima[1], Zhongchuzi Shen[2], Masashi Miura[1], Hideto Nakabayashi[2], Masahiro Kawahara[1,2,*]

[1]Laboratory of Cell Vaccine, Center for Vaccine and Adjuvant Research (CVAR), National Institutes of Biomedical Innovation, Health and Nutrition (NIBIOHN), Ibaraki-shi, Osaka, Japan

[2]Department of Chemistry and Biotechnology, Graduate School of Engineering, The University of Tokyo, Bunkyo-ku, Tokyo, Japan

[*]Corresponding author

E-mail: m-kawahara@nibiohn.go.jp (MK)

METDTLLLWVLLLWVPGSTGDGKPIPNPLLGLDSTGAQPADVVMTQTPLSLPVSLGD

QASISCRSSQSLVHSNGNTYLRWYLQKPGQSPKVLIYKVSNRVSGVPDRFSGSGSGTD

FTLKINRVEAEDLGVYFCSQSTHVPWTFGGGTKLEIKSSADDAKKDAAKKDDAKKD

DAKKDGGVKLDETGGGLVQPGGAMKLSCVTSGFTFGHYWMNWVRQSPEKGLEWV

AQFRNKPYNYETYYSDSVKGRFTISRDDSKSSVYLQMNNLRVEDTGIYYCTGASYG

MEYLGQGTSVTVSGSGVLLDAPVGLVARLADESGHVVLRWLPPPETPMTSHIRYEVD

VSAGNGAGSVQRVEILEGRTECVLSNLRGRTRYTFAVRARMAEPSFGGFWSAWSEPV

SLLTPSDLDPDITALVTSVATVLGAGLVAAGLLLWWRKSLLYRLCPPIPRLRLPLAGEM

VVWEPALEDCEVTPVTDA

S1 Fig. The amino acid sequence of S(M)-IL3Rα.
Grey: leader sequence derived from immunoglobulin κ chain. Light blue: V5 tag. Black: linker sequence. Dark green: FL-specific scFv clone 4M5.3. Yellow: D2 domain of erythropoietin receptor. Pink: transmembrane and intracellular domains of IL3Rα.

METDTLLLWVLLLWVPGSTGDDYKDDDDKIGAQPADVVMTQTPLSLPVSLGDQASIS
CRSSQSLVHSNGNTYLRWYLQKPGQSPKVLIYKVSNRVSGVPDRFSGSGSGTDFTLKI
NRVEAEDLGVYFCSQSTHVPWTFGGGTKLEIKSSADDAKKDAAKKDDAKKDDAKK
DGGVKLDETGGGLVQPGGAMKLSCVTSGFTFGHYWMNWVRQSPEKGLEWVAQFR
NKPYNYETYYSDSVKGRFTISRDDSKSSVYLQMNNLRVEDTGIYYCTGASYGMEYL
GQGTSVTVSGSGVLLDAPVGLVARLADESGHVVLRWLPPPETPMTSHIRYEVDVSAG
NGAGSVQRVEILEGRTECVLSNLRGRTRYTFAVRARMAEPSFGGFWSAWSEPVSLLTP
SDLDPDIVMPTLWIVLILVFLILTLLLILRFGCVSVYRTYRKWKEKIPNPSKSLLFQDGG
KGLWPPGSMAAFATKNPALQGPQSRLLAEQQGESYAHLEDNNVSPLTIEDPNIIRVPPS
GPDTTPAASSESTEQLPNVQVEGPTPNRPRKQLPSFDFNGPYLGPPQSHSLPDLPDQLG
SPQVGGSLKPALPGSLEYMCLPPGGQAQLVPLSQVMGQGQAMDVQCGSSLETSGSPS
VEPKENPPVELSMEEQEARDNPVTLPISSGGPEGSMMASDYVTPGDPVLTLPTGPLST
SLGPSLGLPSAQSPSLCLKLPRVPSGSPALGPPGFEDYVELPPSVSQAAKSPPGHPAPPV
ASSPTVIPGEPREEVGPASPHPEGLLVLQQVGDYCFLPGLGPGSLSPHSKPPSPSLCSET
EDLVQDLSVKKFPYQPMPQAPAIQFFKSLKHQDYLSLPPWDNSQSGKVC

S2 Fig. The amino acid sequence of S(M)-IL3Rβc.

Grey: leader sequence derived from immunoglobulin κ chain. Light green: Flag tag. Black: linker sequence. Dark green: FL-specific scFv clone 4M5.3. Yellow: D2 domain of erythropoietin receptor. Dark blue: transmembrane and intracellular domains of IL3Rβc.

METDTLLLWVLLLWVPGSTGDYPYDVPDYAGAQPADIVMTQSPAIMSASPGEKVTM

TCRASSSVSSTYFHWYQQKSGASPKLWIYSTSTLASGVPARFSGSGSGTSYSLTISSVE

AEDAATYYCQQYSGYPLTFGAGTKLELKRGGGGGSGGGGSGGGGSQVQLQQSGPEL

EKPGASVKISCKASGYSFTGYIMNWVKQNNGKSLEWIGNIAPYYGGTSYNQKFKGK

ATLTVDKSSSTAYMQLSSLTSEDSAVYFCARWGGTMITGLDYWGQGTTLTVSSGSGV

LLDAPVGLVARLADESGHVVLRWLPPPETPMTSHIRYEVDVSAGNGAGSVQRVEILE

GRTECVLSNLRGRTRYTFAVRARMAEPSFGGFWSAWSEPVSLLTPSDLDPDILNIFLGI

LCLVLLSTTCVVTWLCCKRRGKTSFWSDVPDPAHSSLSSWLPTIMTEETFQLPSFWDS

SVPSITKITELEEDKKPTHWDSESSGNGSLPALVQAYVLQGDPREISNQSQPPSRTGDQ

VLYGQVLESPTSPGVMQYIRSDSTQPLLGGPTPSPKSYENIWFHSRPQETFVPQPPNQE

DDCVFGPPFDFPLFQGLQVHGVEEQGGF

S3 Fig. The amino acid sequence of S(U)-GCSFR.
Grey: leader sequence derived from immunoglobulin κ chain. Orange: HA tag. Black: linker sequence. Brown: DNP-specific scFv clone U7.6. Yellow: D2 domain of erythropoietin receptor. Red: transmembrane and intracellular domains of GCSFR.

Antibody	Manufacturer	Cat#
mouse anti-V5 tag	Santa Cruz Biotechnology	sc-18594
mouse anti-HA tag	BioLegend	MMS-101P
mouse anti-Flag tag	Sigma-Aldrich	F1804
PE-conjugated donkey F(ab')2 anti-mouse IgG	Jackson ImmunoResearch	715-116-150

S4 Fig. The antibodies used in cell surface immunostaining.

Antibody	Manufacturer	Cat#
rabbit anti-phospho-STAT3 (Y705)	Cell Signaling Technology	9145
rabbit anti-STAT3	Santa Cruz Biotechnology	sc-482
rabbit anti-phospho-MEK1/2 (S217/221)	Cell Signaling Technology	9154
rabbit anti-MEK1/2	Cell Signaling Technology	8727
rabbit anti-phospho-Akt (Ser473)	Cell Signaling Technology	4060
rabbit anti-Akt	Cell Signaling Technology	9272
HRP-conjugated goat anti-rabbit IgG	Thermo Fisher Scientific	G-21234

S5 Fig. The antibodies used in Western blotting.

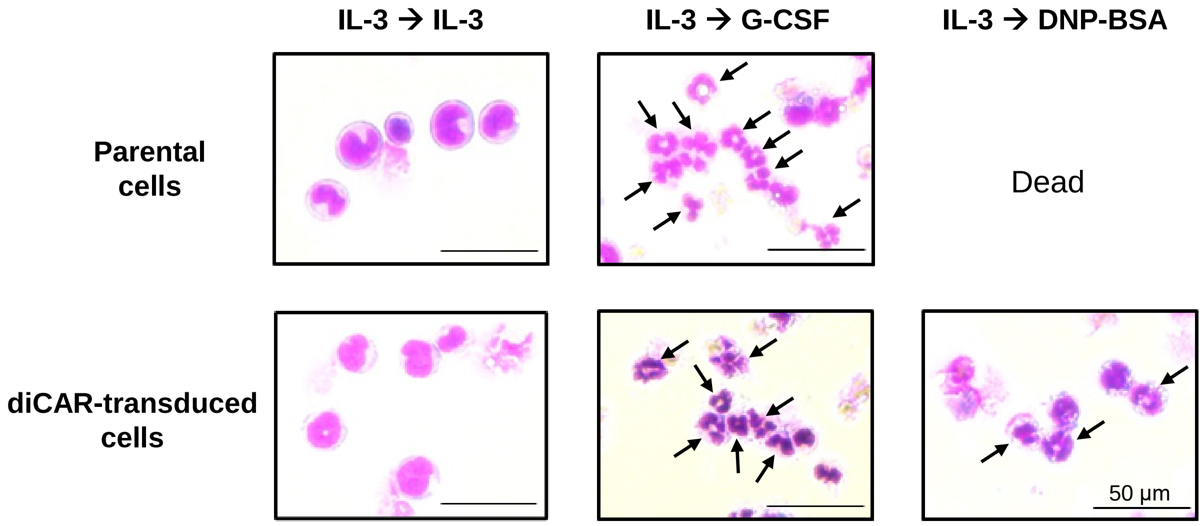

S6 Fig. May-Grünwald-Giemsa staining in the differentiation assay of diCAR-transduced cells.

Cells precultured with 1 ng/ml IL-3 were washed and cultured with 1 ng/ml IL-3, 10 ng/ml G-CSF, or 0.1 µg/ml DNP-BSA. On day 5, the cells were stained with the staining solutions. Arrows in the photographs indicate granulocytic cells with segmented nuclei. Scale bar: 50 µm

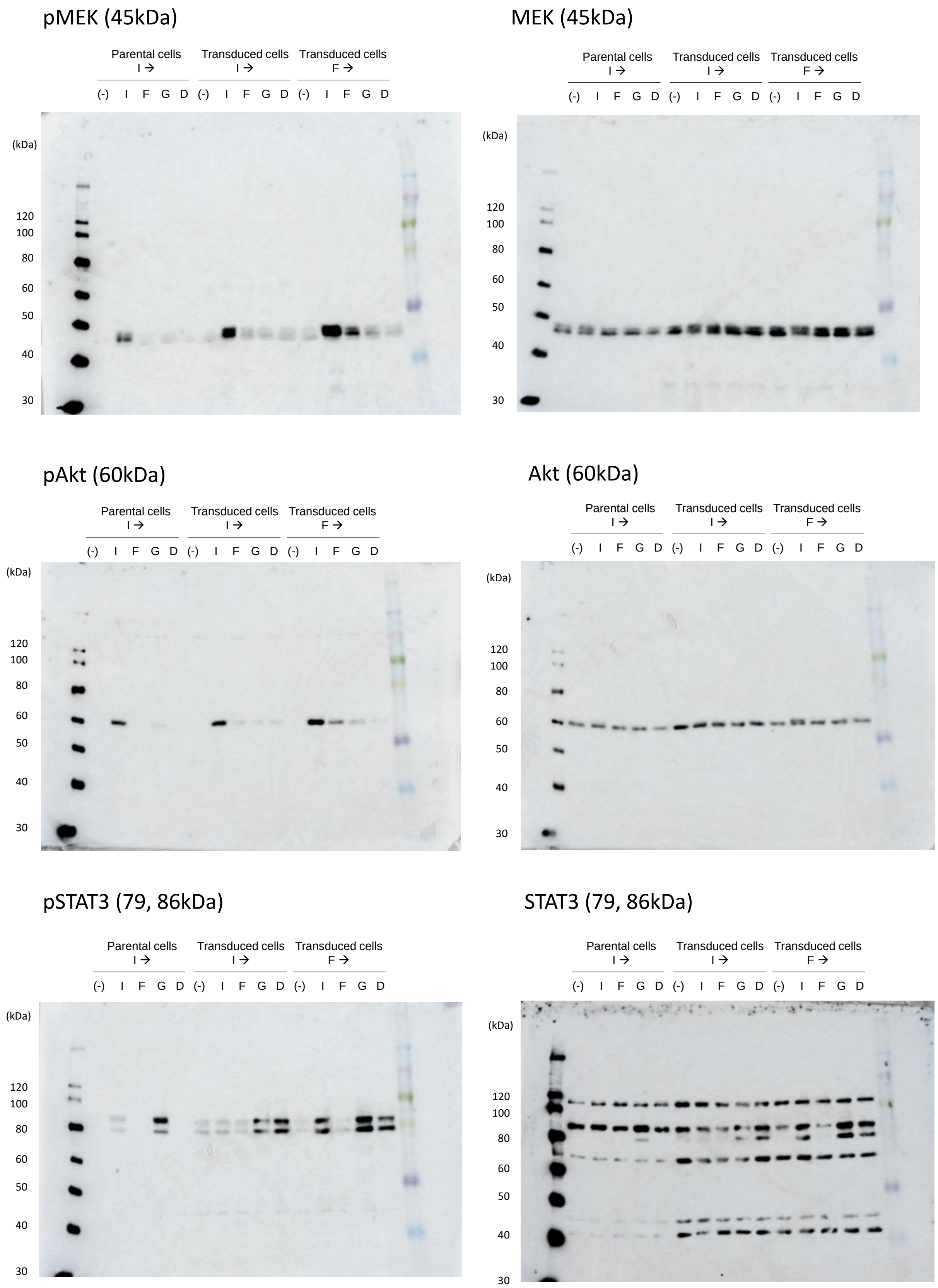

S7 Fig. Uncropped blot images.
